# Similar Muscle Recovery but Greater Soreness in Active Vegans on Low‐Protein Diets Compared to High‐Protein Omnivores Following Exercise‐Induced Damage

**DOI:** 10.1111/nbu.70064

**Published:** 2026-08-12

**Authors:** Raquel Simões Mendes‐Netto, Felipe Garcez de Carvalho, Alice Conrado de Souza, Marcos da Silva Brandão, David Lima Oliveira, Marcela Larissa Costa, Marcus Vinicius Santos do Nascimento, Marzo Edir da Silva Grigoletto

**Affiliations:** ^1^ Postgraduate Program in Physical Education Federal University of Sergipe São Cristóvão Sergipe Brazil; ^2^ Postgraduate Program in Nutrition Sciences Federal University of Sergipe São Cristóvão Sergipe Brazil; ^3^ Nutrition Department Federal University of Sergipe São Cristóvão Sergipe Brazil; ^4^ Department of Nutrition Tiradentes University Aracaju Sergipe Brazil

**Keywords:** diet, muscle damage, muscle recovery, plant‐based diet, veganism

## Abstract

To assess whether a low‐protein vegan diet is associated with differences in recovery from exercise‐induced muscle damage (EIMD) in physically active individuals. Physically active vegans (*n* = 7) and omnivores (*n* = 7) underwent a lower‐body muscle damage protocol consisting of five sets of repetitions to failure using the Smith machine squat and leg press at 85% of one‐repetition maximum (1RM). Muscle damage and recovery were assessed at baseline, immediately post‐exercise, and at 24, 48, and 72 h post‐EIMD. Outcomes included serum concentrations of creatine kinase (CK) and lactate dehydrogenase (LDH), delayed‐onset muscle soreness (DOMS), countermovement jump (CMJ) performance, and range of motion (ROM). Habitual dietary intake was assessed using four 24‐h dietary recalls analysed via the Multiple Source Method (MSM) software. Participants demonstrated similar training volume across exercises and comparable hydration status. Protein intake was significantly lower in vegans (0.98 g/kg) compared to omnivores (2.14 g/kg), along with reduced intake of essential amino acids and, specifically, branched‐chain amino acids (*p* ≤ 0.05). CK levels increased immediately after EIMD and peaked at 24 h, remaining elevated relative to baseline in both groups (*p* ≤ 0.05). LDH levels rose immediately post‐exercise in both groups and were still elevated at 24 h (*p* ≤ 0.05). DOMS was markedly greater in vegans (+2.7 at 24 h; +2.6 at 72 h on VAS) than in omnivores at 24 and 72 h post‐exercise (*p* ≤ 0.05). While habitually active vegans and omnivores exhibited similar overall responses in muscle damage and recovery following intense resistance exercise, vegans experienced clinically meaningful increases in DOMS. These findings suggest that a low‐protein vegan diet may be associated with increased perceived muscle damage, without impairing acute functional recovery. For individuals following a vegan diet, these results highlight the importance of ensuring adequate protein intake and protein quality, including sufficient essential amino acids and branched‐chain amino acids, to potentially mitigate exercise‐induced muscle soreness and optimise recovery comfort.

## 1 Introduction

The vegan diet is being increasingly adopted by different populations (Cramer et al. 2017; Pelly and Burkhart 2014; Ploll et al. 2020; Turner‐McGrievy et al. 2016). It is characterised by the exclusion of foods of animal origin, leading to differences in nutrient intake compared with an omnivorous diet, which includes both plant and animal foods (Melina et al. 2016). It has been consistently reported that individuals following a vegan diet have higher intakes of vegetables, legumes, and fruits than their omnivorous counterparts, and this dietary pattern is considered healthy (Clarys et al. 2013, 2014).

Previous studies have shown that vegans and vegetarians have better or similar aerobic capacity compared with omnivores (Boutros et al. 2020; Nebl et al. 2019). However, evidence regarding strength and power outcomes remains less conclusive. A systematic review suggested that vegan and vegetarian dietary patterns, including lacto‐ovo‐vegetarian diets, do not impair strength or power performance (Damasceno et al. 2024), whereas another systematic review focusing on vegetarians suggests potential improvements in countermovement jump (CMJ) performance and relative strength among healthy trained adults (Presti, Mansouri, et al. 2024). Beyond performance outcomes, muscle damage and recovery should also be considered, as they are relevant to muscle integrity and athletic performance.

Muscle damage occurs when the stress of intense or unfamiliar exercise disrupts muscle fibres or when exercise involves eccentric contractions (Mathur et al. 2010). This process increases the breakdown of muscle fibres and leads to the release of cytosolic proteins such as creatine kinase (CK) and lactate dehydrogenase (LDH). These proteins are normally confined to muscle cells. However, they are released into the circulation following muscle damage and are commonly used as biomarkers of exercise‐induced muscle damage (EIMD) (Koch et al. 2014). EIMD can also lead to delayed‐onset muscle soreness (DOMS), muscle swelling, and reduced range of motion (ROM) (Clarkson and Sayers 1999; Howatson and van Someren 2008). These symptoms can significantly limit subsequent physical performance by reducing the individual's ability to exercise at full intensity, impairing movement efficiency, and increasing the risk of injury (Cheung et al. 2003).

Vegan diets may influence muscle damage and recovery following intense exercise. A vegan diet high in micronutrients, as well as phytochemicals and polyphenols, may aid post‐exercise muscle recovery due to their antioxidant and anti‐inflammatory effects (Harty et al. 2019; Jakeman et al. 2017; McLeay et al. 2012; Shirato et al. 2016). However, lower protein intakes reported in vegan populations may lead to insufficient muscle recovery after strenuous exercise, given the essential role of protein in post‐exercise muscle repair (Neufingerl and Eilander 2021). As protein is critical for muscle function, inadequate intake may impair recovery and reduce protection against EIMD (Brown et al. 2018; Elorinne et al. 2016; Schmidt et al. 2016; Shirato et al. 2016). Increasing protein intake—particularly essential and branched‐chain amino acid intake—alongside adequate micronutrient intake is an effective strategy to reduce post‐exercise muscle soreness, mitigate muscle swelling and range of motion limitations, and promote the recovery of muscle function after damage (Church et al. 2020; Rahimi et al. 2017).

It is important to note that when protein intake is matched, vegan and omnivorous diets show no differences in strength measures following resistance training programs, as demonstrated at intakes of ~1.6 g/kg/day (Hevia‐Larraín et al. 2021) and ~2.0 g/kg/day (Monteyne et al. 2023). To our knowledge, only one study has directly investigated the effect of habitual diet on post‐EIMD recovery. Presti, Rideout, et al. (2024) reported that individuals consuming an omnivorous diet recovered more quickly than vegetarians following an EIMD protocol induced by repeated eccentric contractions of the quadriceps, with recovery assessed over five days through changes in isometric and concentric strength, vertical jump performance, and muscle soreness. The authors attributed this finding to higher protein intake and the greater bioavailability of animal‐based proteins. Additionally, assessing recovery under habitual dietary conditions, rather than prescribed protein intake protocols, provides a more realistic view of real‐world eating behaviours and their combined effects on muscle recovery.

Although these findings are significant, it is important to note that the study did not include individuals who habitually followed a strict vegan diet. Given that a habitual vegan diet may provide micronutrients beneficial for recovery while simultaneously providing lower protein intake than vegetarian and omnivorous diets (Neufingerl and Eilander 2021), which could delay recovery. There is currently no published study, to the best of our knowledge, that has evaluated the impact of habitual dietary intake on muscle recovery following muscle damage in vegans compared with omnivores. Thus, we hypothesise that lower habitual protein and essential amino acid intake in vegans may impair acute muscle recovery following EIMD, despite the potential benefits of higher micronutrient intake. Therefore, the present study aimed to compare the recovery of muscle function, DOMS, ROM, muscle swelling, and the attenuation of CK and LDH release following EIMD in physically active vegans and omnivores engaged in resistance training.

## 2 Methods and Materials

### 2.1 Experimental Design

**FIGURE 1:**
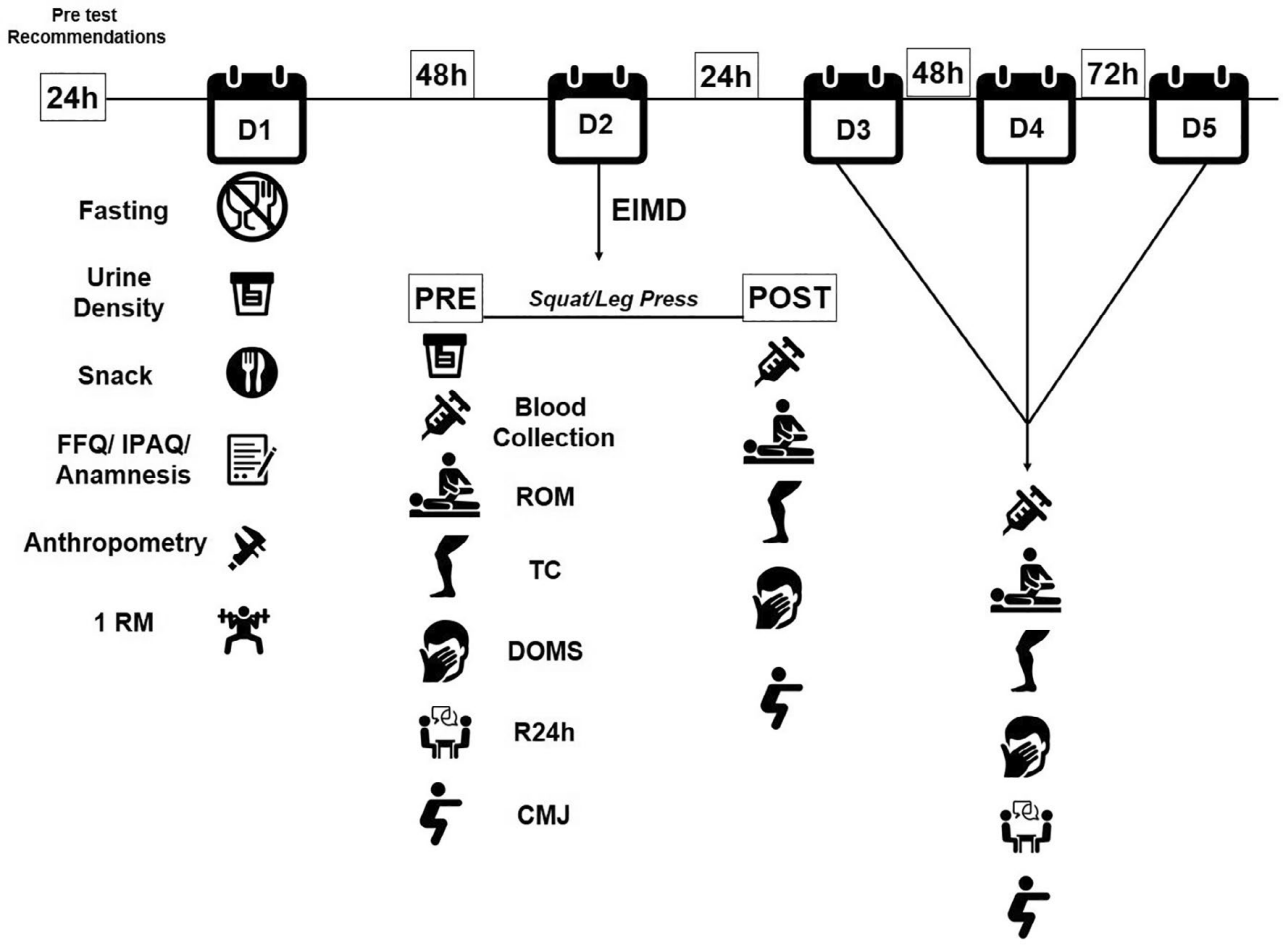
Study design. Study took place in 5 meetings (D1, D2, D3, D4, and D5). One participant was excluded from the analysis due to being a high responder, which was deemed to potentially skew the overall data interpretation. This exclusion is indicated in the figure. 1RM, maximum dynamic strength assessed by the 1 repetition maximum test; CMJ, muscle power assessed by jumping with counter movement; CT, Thigh Circumference; DOMS, late muscular pain; FFQ, Food frequency questionnaire; IPAQ, International Physical Activity Questionnaire; R24h, 24‐h recall; ROM, range of motion.

This study was conducted among physically active vegan and omnivorous individuals. Five visits were required for dietary and anthropometric assessment, protocol familiarisation, physical test application, and muscle recovery analysis. Figure 1 shows the experimental design according to the execution order of the protocols.

Participants were instructed to sleep for at least 7 h on the night before each testing session (1RM test and EIMD protocol); to not use any sports nutrition supplements (e.g., protein powder, creatine, beta‐alanine, or pre‐workout formulas) for at least 1 month before and throughout the study period. The use of essential micronutrient supplements (e.g., vitamin B12 and iron) was neither restricted nor systematically assessed. Participants were also instructed not to consume caffeinated foods, energy drinks, or alcohol; to drink water the day before testing (35 mL/kg of body weight); and not to exercise during the 48 h before testing or throughout the entire collection period. On the day of the 1RM test, participants arrived in a fasted state and consumed a snack (552.3 kcal; 126.2 g of carbohydrate; 8.3 g of protein; and 3 g of lipid) to standardise their pre‐exercise meal conditions for the 1RM test. Hydration status was also analysed by urine specific gravity on the day of the 1RM test.

### 2.2 Participants

The sample size was estimated using G*Power 3.1 (Faul et al. 2009), following procedures recommended by Beck (2013) for repeated‐measures ANOVA designs (Beck 2013). Serum CK at 24 h post‐exercise was designated as the primary outcome for this calculation, as CK typically peaks at this time point following lower‐body resistance exercise and represents the most sensitive and widely used indirect marker of EIMD (Brancaccio et al. 2007; Nosaka and Clarkson 1996). The following parameters were applied: power (1 − *β*) = 0.80; *α* = 0.05; effect size *f* = 0.71 (derived from: Callegari et al. 2017), correlation among repeated measures = 0.5; non‐sphericity correction *ε* = 1. The analysis indicated that a minimum of six participants per group was required.

The study included vegan and omnivorous individuals who met the inclusion criteria described below. The sample was selected by convenience, with recruitment conducted through calls on social media platforms (i.e., Facebook and Instagram), posters, and institutional emails sent to faculty, technicians, and students at a public university in Northeast Brazil. The inclusion criteria were: age 18–35 years, to have practiced resistance exercise for at least 12 months at an average frequency of ≥ 3 sessions per week; to have adhered to their respective diet (vegan or omnivorous) for at least 6 months; to not have regularly used sports nutrition supplements in the six months prior to study enrollment (see Table S1 for all sports nutrition supplements considered in the present study); to not be current or former smokers (i.e., individuals who have never smoked); and to not have experienced any musculoskeletal injury in the lower extremities within the six months prior to enrollment that could limit performance of the squat or leg press exercises or affect lower‐limb muscle damage and recovery responses. Participants who reported prior use of sports nutrition supplements completed a 30‐day washout period before commencing the study protocol—see Washout Protocol below. Micronutrient supplements used for health maintenance (e.g., vitamin B12, iron, vitamin D) were not restricted.

**FIGURE 2:**
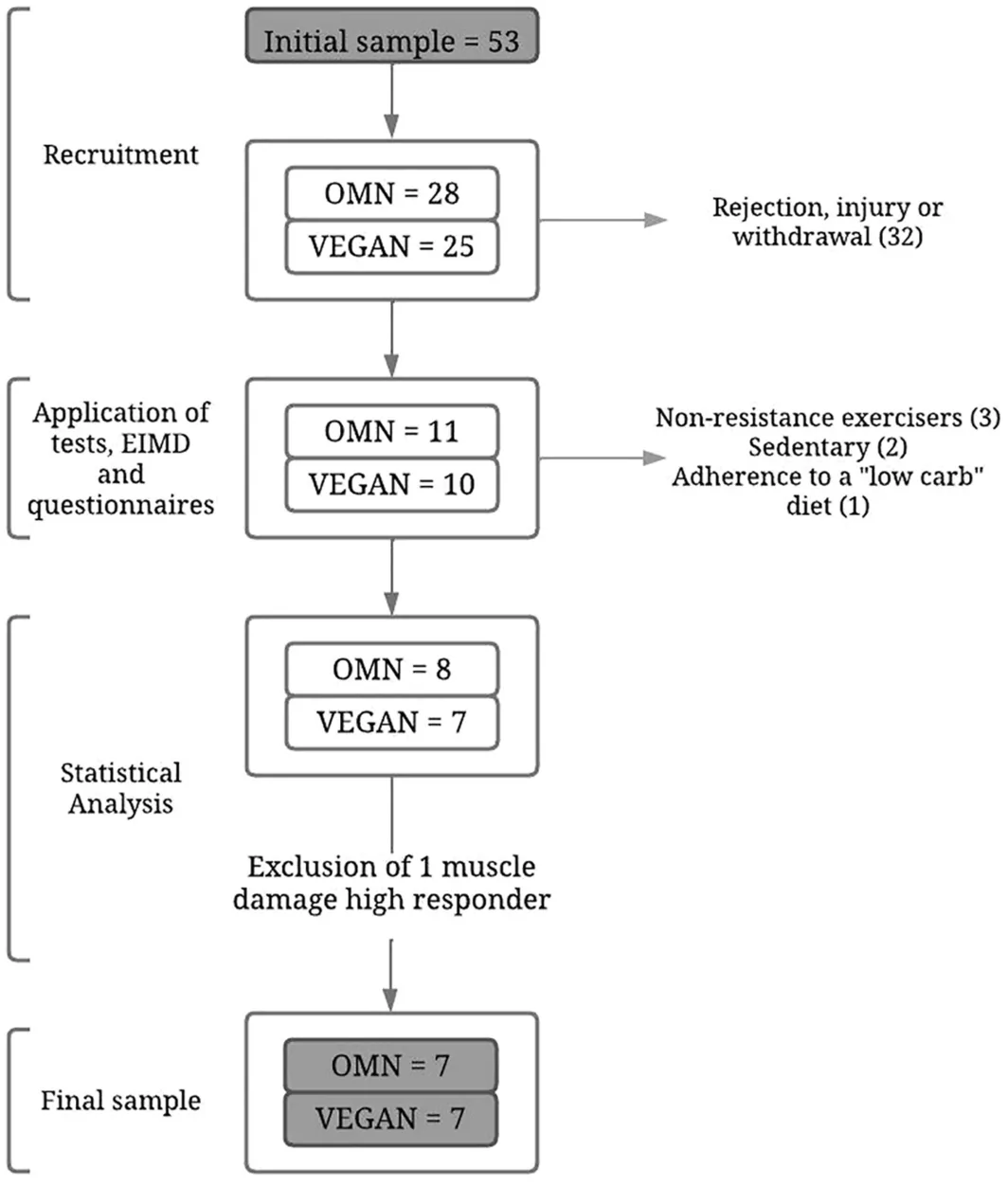
Flowchart from sample selection to obtaining the final *n*. EIMD, exercise‐induced muscle damage; OMN, Omnivores; VEGAN, Vegans.

For vegan participants, adherence was further confirmed through a detailed dietary interview to ensure strict avoidance of all animal products. Individuals who did not fit into either dietary group, those who followed a specific diet that could interfere with the study results (i.e., a low‐carbohydrate diet), and individuals who performed exclusively aerobic exercise were excluded. After evaluation of the inclusion and exclusion criteria, the study included seven vegans and seven omnivores (Figure 2). During the selection process, participants completed the short version of the International Physical Activity Questionnaire (IPAQ), validated for the Brazilian population (Matsudo et al. 2001) to estimate their physical activity levels. Additional information regarding exercise experience was also collected.

To confirm adherence to the self‐reported dietary group, participants completed a Food Frequency Questionnaire (FFQ) developed by the authors. This FFQ included five animal‐based food items to assess the consumption of animal‐derived foods over the previous six months. For vegan participants, adherence was further confirmed through a detailed dietary interview to ensure strict avoidance of all animal products. The FFQ analysis was confirmatory, primarily verifying the frequency of consumption of animal‐origin foods, nutritional supplements, milk, and dairy products, and assessing whether these frequencies aligned with participants' self‐classification of their dietary group.

To minimise potential confounding from prior supplement use on muscle damage and recovery biomarkers, a 30‐day washout period from sports nutrition supplementation was required before study commencement. This duration was selected based on evidence that, once intramuscular creatine stores are elevated, it generally takes 4–6 weeks for stores to return to pre‐supplementation baseline following cessation (Kreider et al. 2017). The 30‐day period therefore represents a conservative minimum consistent with this evidence. During this period, participants were instructed to completely abstain from using any performance‐enhancing or sports nutrition supplements (e.g., protein powder, creatine, or beta‐alanine). This protocol was applied to all participants in both groups. It is important to note that this washout protocol specifically targeted sports supplements and did not restrict the use of micronutrient supplements (e.g., vitamin B12 and iron), which were not assessed in this study.

In compliance with the Helsinki Declaration, the study was approved by the Institutional Review Board of the Federal University of Sergipe (CEP‐UFS; CAAE approval number: 2.321.614). All participants provided written informed consent prior to participation in the study, including the right to withdraw at any time without consequence.

### 2.3 Measures

#### 2.3.1 Urine Density

Urine specific gravity (USG) was measured on the day of the 1RM test and on the day of the EIMD protocol to verify comparable hydration status between and within groups. Participants collected a midstream urine sample upon arrival at the laboratory for each session. USG was measured using a calibrated refractometer available at the Exercise Physiology Laboratory of the Federal University of Sergipe, Brazil. Participants were classified as adequately hydrated if USG was ≤ 1.020 or mildly dehydrated if USG was 1.021–1.030, following standard hydration classification criteria (Armstrong et al. 1994). Any participant presenting with severe dehydration (USG > 1.030) was asked to rehydrate before testing.

#### 2.3.2 Anthropometric Measures

Body mass was measured using a calibrated digital scale (P‐150M, Líder, Brazil), and stature was measured using a portable stadiometer (ALTURAEXATA, Brazil). Body mass index (BMI) was calculated from these measurements. Body composition was estimated from skinfold measurements using the Petroski (1999) equations for body density, from which fat percentage (F%) and lean mass (LM) were derived (Petroski 1999). Sex‐specific skinfold sites were measured: subscapular, triceps, suprailiac, and calf skinfolds in men; and midaxillary, suprailiac, thigh, and calf skinfolds in women. All skinfold measurements were performed in triplicate by a single trained evaluator using a calibrated skinfold calliper, with a coefficient of variation ≤ 5% required for acceptance.

#### 2.3.3 Dietary Assessment

Dietary intake was assessed using four non‐consecutive 24‐h dietary recalls (24HRs). These were administered on: (1) the day of the EIMD protocol; (2) 24 h post‐EIMD; (3) 48 h post‐EIMD; and (4) 72 h post‐EIMD, thus capturing dietary intake during the full recovery monitoring period. Recalls were conducted using the multiple‐pass method to maximise the accuracy of reported food intake (Blanton et al. 2006).

Dietary data were tabulated using NDSR software (Nutrition Data System for Research, version 2014, University of Minnesota), supplemented by Brazilian food composition reference tables (Instituto Brasileiro de Geografia e Estatística 2011; Nepa 2011) and a standardised household measures guide (Instituto Brasileiro de Geografia e Estatística 2011). Food portions reported in household measures were converted to grams (g) or millilitres (mL) using these references, and all nutrient values—energy in kcal, macronutrients in g, micronutrients in mg or μg, and amino acids in g—were derived from the software output. The Multiple Source Method (MSM) (Harttig et al. 2011) was then applied to the four 24‐h recalls to model habitual dietary intake for each participant.

Diet quality was evaluated using the Healthy Eating Index‐2015 (HEI‐2015) (Krebs‐Smith et al. 2018) a validated tool designed to assess alignment of dietary intake with the 2015–2020 Dietary Guidelines for Americans. The HEI‐2015 was applied to the habitual dietary intake data derived from the four 24‐h recalls after the MSM step to characterise and compare overall diet quality between vegans and omnivores. Scores range from 0 to 100, with higher scores reflecting greater alignment with dietary recommendations. The HEI‐2015 was selected as a secondary dietary outcome to complement the macronutrient and micronutrient analyses, allowing a broader characterisation of dietary quality in each group. Energy requirements were estimated using data on sex, age, weight, height, and physical activity level. These variables were applied to the equations proposed in the 2023 Dietary Reference Intakes (DRIs) to calculate the Estimated Energy Requirement (EER) (National Academies of Sciences Engineering and Medicine 2023).

#### 2.3.4 Muscle Damage Protocol

##### 2.3.4.1 One‐Repetition Maximum Test (1RM)

The one‐repetition maximum (1RM) test was applied to two exercises: the guided‐barbell squat (Smith machine) and the 45° leg press. The 45° leg press is a machine‐based lower‐limb exercise in which participants push a weighted platform along a fixed 45‐degree inclined track by extending the knees and hips, returning to the starting position under controlled eccentric loading. Both exercises were performed on equipment available at the Exercise Physiology Laboratory of the Federal University of Sergipe, Brazil.

Before the test, participants performed a warm‐up set of 10 repetitions at approximately 50% of their self‐reported estimated maximum load, based on their training experience with each exercise. After a 3‐min rest, the test commenced with an initial load selected by the evaluator based on the participant's training history and warm‐up performance. For each attempt, participants were required to complete a single full repetition. If successful, the load was increased by 2.5–5 kg, and another attempt was made after a 3–5‐min rest interval (Clarke 1973). If an attempt failed, the load was reduced by the same increment for a subsequent attempt. Participants were permitted a maximum of six attempts in total to minimise cumulative muscular fatigue and avoid compromising subsequent assessments. The 1RM was defined as the greatest load successfully lifted through the full range of motion with correct technique. All movements were performed at a cadence of 2 s for the concentric phase and 4 s for the eccentric phase, with range of motion standardised for both the Smith machine squat and the 45° leg press according to previous recommendations (Baroni et al. 2017). Relative strength was calculated by dividing the 1RM load for each exercise by body mass. The individuals performed 10 repetitions with a load of 50% of the perceived maximum repetition before starting the test for familiarisation on each piece of equipment. Then, the test was initiated, and the participants made one attempt to complete one repetition with maximum load.

##### 2.3.4.2 Exercise‐Induced Muscle Damage (EIMD)

The EIMD protocol was prescribed at an intensity of 85% of 1RM for the Smith machine squat and the 45° leg press. All participants started with a warm‐up set without additional load, performing 10 repetitions of the Smith machine squat. Participants then performed five sets of squat repetitions to concentric failure and five sets on the 45° leg press, with a movement cadence of 2 s for the concentric phase and 4 s for the eccentric phase, and 2‐min recovery intervals between sets. The number of repetitions in each set was recorded to calculate the total training volume in each group. Range of motion was standardised consistently across both the 1RM test and the EIMD protocol: squat depth was controlled to achieve approximately 90° of knee flexion, and the leg press range of motion was set from full knee extension to approximately 90° of knee flexion, following previous recommendations (Baroni et al. 2017; Hasenoehrl et al. 2017).

#### 2.3.5 Muscle Damage Measures

All muscle damage outcomes were assessed at the time points described in Figure 1: pre‐EIMD, immediately post‐EIMD, and 24, 48, and 72 h post‐EIMD.

##### 2.3.5.1 Blood Collection

For analysis of CK and LDH enzymes, 7 mL of blood was collected in a gel separator tube, which was then centrifuged within 10 min of collection. Serum was then aliquoted into Eppendorf tubes, and the samples for CK analysis were protected from light. The samples were analysed using a BioPlus 2000 semi‐automatic analyser, with procedures standardised using Labtest analysis kits. All procedures were performed in triplicate, adopting a coefficient of variation of 5%.

##### 2.3.5.2 Delayed‐Onset Muscle Soreness (DOMS)

Longitudinal and transverse palpation was performed in the quadriceps muscle group to assess perceived pain after exercise. Participants also completed a visual analog scale (VAS) for pain (Lau et al. 2015). Participants were instructed to recall the greatest pain they had ever felt and use it as a reference.

#### 2.3.6 Muscle Recovery Measures

All muscle recovery outcomes followed the same assessment schedule described in Figure 1: pre‐EIMD, immediately post‐EIMD, and 24, 48, and 72 h post‐EIMD.

##### 2.3.6.1 Countermovment Jump (CMJ)

Lower‐body power was evaluated using the CMJ (Komi and Bosco 1978). Participants were instructed to perform three vertical jumps preceded by a squat to 90° of knee flexion and then jump vertically with maximum effort. No specific warm‐up was performed before CMJ assessment at any time point, as a warm‐up could confound recovery status measurements. Participants were instructed to rest for at least 10 min after any locomotion or standing activity before testing. The highest value among the three attempts was used for data analysis.

##### 2.3.6.2 Range of Motion (ROM)

Range of motion was evaluated using the protocol described by Batista et al. (2006). Participants were placed in a supine position and asked to flex their knees and hips at 90° while keeping their feet relaxed. The evaluator then extended the knee from this position without allowing the participant to perform any contraction. The final position was defined as the moment when the participant felt the onset of knee flexor muscle tension, measured with a universal goniometer (Batista et al. 2006). The difference between the initial and final positions was calculated to analyse the degree of knee flexor shortening, and the average of three measurements was used in the data analysis.

##### 2.3.6.3 Thigh Circumference (TC)

Thigh circumference was measured at the midpoint of the right thigh as a proxy for muscle swelling, following the protocol of Batista et al. (2006). The midpoint was defined as the point equidistant between the greater trochanter of the femur and the lateral joint line of the knee and was marked with a skin marker to ensure consistent placement across all assessment time points. Measurements were taken with participants in a standing position with weight equally distributed between both legs, using a flexible, non‐elastic anthropometric tape measure (Batista et al. 2006). Three consecutive measurements were recorded, and the mean was used for data analysis, adopting a coefficient of variation ≤ 5%.

#### 2.3.7 Analysis

Statistical analysis was performed using SPSS version 28.0. Continuous variables were subjected to the Shapiro–Wilk test to verify the assumption of normality. One participant was excluded from the final analysis after being identified as a statistical outlier based on pre‐established criteria, defined as values exceeding 3 standard deviations from the mean for the outcomes. This exclusion was necessary to ensure that the results accurately represented the typical physiological response of the study sample, as this participant's extreme values disproportionately skewed group means and variances. The consistency of this aberrant response across all post‐exercise measurements (24, 48, and 72 h) confirmed that it was not a measurement error but rather represented a genuinely atypical physiological response to the protocol.

A descriptive analysis was performed using absolute numbers and percentages for categorical variables and mean and standard deviation for continuous variables. Pearson's chi‐squared or Fisher's exact test was performed to verify associations between categorical descriptive variables. Comparisons of urine density between the days of the 1RM test and the EIMD test, as well as differences between energy requirement and energy intake, were conducted using paired *t*‐tests or Wilcoxon's tests, as appropriate. Student's *t*‐test or Mann–Whitney *U*‐test were used for parametric and non‐parametric variables, respectively, to verify the comparison between age, dietary intake, and anthropometric profile between the eating pattern groups. Finally, the analysis of muscle damage and recovery measures in interaction with the dietary groups was performed using Factorial ANOVA for repeated measures (time × dietary group) with Bonferroni post hoc, adopting the relative strength of the leg 45° and the squat as covariates. The assumption of sphericity was assessed using Mauchly's test. When this assumption was violated, degrees of freedom were corrected using the Greenhouse–Geisser correction. Partial eta squared ($\eta_{p}^{2}$) was calculated to measure ES at the ANOVA, with thresholds of 0.01, 0.06, and 0.14 considered small, medium, and large, respectively (Cohen 1988; Richardson 2011). Cohen's *d* was provided for ES of post hoc comparisons, with thresholds of 0.2, 0.6, 1.2, and 2.0 considered trivial, small, moderate, large, and very large, respectively (Hopkins et al. 2009). A *p*‐value of < 0.05 was considered significant.

## 3 Results

Table 1 presents the descriptive characteristics of the participants. The final sample comprised 14 participants: seven vegans and seven omnivores. Age did not differ between omnivores and vegans (24.57 ± 3.04 vs. 25.71 ± 5.85 years; *p* = 0.655). Body weight (63.35 ± 10.47 vs. 61.20 ± 3.30 kg; *p* = 0.613), height (166.14 ± 9.99 vs. 173.85 ± 3.43 cm; *p* = 0.092), fat mass (10.61 ± 3.19 vs. 8.78 ± 4.15 kg; *p* = 0.238), and lean mass (52.88 ± 9.35 vs. 52.41 ± 6.21 kg; *p* = 0.337) were also similar between groups. BMI was higher in omnivores than in vegans (22.85 ± 2.00 vs. 20.34 ± 1.51 kg/m^2^; *p* = 0.021).

**TABLE 1:** Sample profile and comparison of variables interfering with performance of the muscle damage induction protocol between healthy active adults, omnivorous and vegans (*n* = 14).

Variables	ONM *n* = 7 (SD)	VEGAN *n* = 7 (SD)	*p‐value*
Age (years)^a^	24.57 ± 3.04	25.71 ± 5.85	0.655
Weight (kg)^a^	63.35 ± 10.47	61.2 ± 3.30	0.613
Height (cm)^a^	166.14 ± 9.99	173.85 ± 3.43	0.092
BMI (kg/m^2^)^a^	22.85 ± 2.00	20.34 ± 1.51	0.021*
FM (kg)^a^	10.61 ± 3.19	8.78 ± 4.15	0.238
LM (kg)^a^	52.88 ± 9.35	52.41 ± 6.21	0.337
Relative strength leg 45°^a^	4.54 ± 0.63	3.39 ± 8.78	0.011*
Relative squat strength^a^	1.39 ± 0.18	1.01 ± 0.35	0.026*
Number of repetitions leg 45°^a^	26 ± 10	32 ± 5	0.167
Number of repetitions squat^a^	30 ± 3	37.42 ± 17	0.331
Urine density (g/cm^3^)—D1^a^	1020.71 ± 9.53	1017.71 ± 6.02	0.495
Urine density (g/cm^3^)—D2^a^	1014.86 ± 11.61	1012.86 ± 4.45	0.339
Sex^b^			
Male	4	5	0.500
Female	3	2	
Time of practice (years)^c^			
1–2	2	1	0.487
> 2	5	6	
Type of exercise (*n*)^c^			
Strength	0	1	0.500
Strength and endurance	7	6	
EER × kcal intake^d^			
EER	2621.65 (147.95)	2672.49 (202.05)	0.937
kcal	2615.35 (756.70)	2371.34 (484.17)	0.297

Abbreviations: EER, estimated energy requirement; FM, fat mass; kcal, kilocalories; LM, lean mass; OMN, omnivore; VEGAN, vegan.

^a^ Student's *t*‐test.

^b^ Chi‐squared test.

^c^ Fischer's exact.

^d^ Wilcoxon signed‐rank test.

* Statistical difference between OMN and VEGAN.

Regarding exercise‐related variables, omnivores had higher relative strength in the 45° leg press than vegans (4.54 ± 0.63 vs. 3.39 ± 0.78; *p* = 0.011) and higher relative squat strength than vegans (1.39 ± 0.18 vs. 1.01 ± 0.35; *p* = 0.026). However, the number of repetitions performed during the EIMD protocol did not differ significantly between omnivores and vegans for either the 45° leg press (26.28 ± 10.17 vs. 32.71 ± 5.49 repetitions; *p* = 0.167) or the squat exercise (30.57 ± 3.64 vs. 37.42 ± 17.53 repetitions; *p* = 0.331). Urine specific gravity was also similar between omnivores and vegans on D1 (1020.71 ± 9.53 vs. 1017.71 ± 6.02 g/cm^3^; *p* = 0.495) and D2 (1014.86 ± 11.61 vs. 1012.86 ± 4.45 g/cm^3^; *p* = 0.339), indicating comparable hydration status between groups on both testing days.

Sex distribution did not differ between groups, with four men and three women among omnivores and five men and two women among vegans (*p* = 0.500). Training experience was also similar between groups: two omnivores and one vegan had 1–2 years of practice, whereas five omnivores and six vegans had more than 2 years of practice (*p* = 0.487). Most participants reported combined strength and endurance training, with seven omnivores and six vegans in this category (*p* = 0.500). Estimated energy requirement was similar between omnivores and vegans (2621.65 ± 147.95 vs. 2672.49 ± 202.05 kcal/day; *p* = 0.937), and reported energy intake did not differ significantly between groups (2615.35 ± 756.70 vs. 2371.34 ± 484.17 kcal/day; *p* = 0.297). Regarding supplement use, one participant initially reported sporadic use of a plant‐based protein supplement, defined as less than once per week, and successfully completed the washout period prior to enrollment; all other participants confirmed no use of such supplements prior to the study.

### 3.1 Dietary Intake

Dietary intake data are presented in Table 2. Total energy intake did not differ significantly between omnivores and vegans (2615.34 ± 756.69 vs. 2371.33 ± 484.17 kcal/day; *p* = 0.488). Absolute carbohydrate intake was also similar between groups (313.56 ± 74.23 vs. 375.51 ± 97.65 g/day; *p* = 0.206), but vegans consumed a higher percentage of energy from carbohydrates than omnivores (63.15 ± 5.56 vs. 48.68% ± 4.76% of energy; *p* ≤ 0.001). Carbohydrate intake relative to body mass did not differ significantly between groups (4.96 ± 0.91 vs. 6.14 ± 1.63 g/kg/day; *p* = 0.121). Total fibre intake was higher in vegans than in omnivores (43.78 ± 9.36 vs. 23.11 ± 10.84 g/day; *p* = 0.026).

**TABLE 2:** Dietary profile among healthy active adults omnivorous and vegans (*n* = 14).

Variables	OMN *n* = 7 (SD)	VEGAN *n* = 7 (SD)	*p‐value*
Total calories (kcal)^a^	2615.00 ± 756.00	2371.00 ± 484.00	0.488
Carbohydrates (g)^a^	313.56 ± 74.23	375.51 ± 97.65	0.206
(%E)^a^	48.68 ± 4.76	63.15 ± 5.56	≤ 0.001^&^
(g/kg)^a^	4.96 ± 0.91	6.14 ± 1.63	0.121
Total fibre (g)^a^	23.11 ± 10.84	43.78 ± 9.36	0.026^&^
Protein (g)^a^	135.11 ± 47.35	60.12 ± 15.15	0.005^&^
(%E)^a^	20.48 ± 2.55	10.02 ± 2.07	≤ 0.001^&^
(g/kg)^a^	2.14 ± 0.63	0.98 ± 0.24	0.002^&^
Vegetal (g)^a^	32.25 ± 8.59	65.08 ± 24.56	0.017^&^
Animal (g)^b^	88.73 ± 67.75	0.00 ± 0.00	≤ 0.001^&^
Essential amino acids (g)^b^	49.30 ± 17.30	18.67 ± 4.16	0.003^&^
Branched chain amino acids (g)^b^	23.73 ± 8.25	9.26 ± 2.05	0.005^&^
Total fats (g)^b^	89.89 ± 32.68	70.78 ± 10.34	0.183
(%E)^a^	28.64 ± 7.76	25.60 ± 8.01	0.128
*Trans*. (g)^a^	2.00 ± 2.62	0.58 ± 0.95	0.038^&^
Saturated fatty acids (g)^a^	33.46 ± 12.59	16.74 ± 2.87	0.012^&^
(%E)^a^	11.55 ± 2.81	6.51 ± 1.35	0.002^&^
Monounsaturated fatty acids (g)^a^	29.45 ± 8.78	26.16 ± 3.34	0.381
(%E)^a^	10.12 ± 0.43	10.19 ± 1.89	0.930
Polyunsaturated fatty acids (g)^a^	17.80 ± 8.77	22.58 ± 4.85	0.047^&^
(%E)^a^	5.83 ± 1.32	8.62 ± 1.03	0.237
Omega‐3 (g)^a^	1.41 ± 1.66	2.42 ± 2.32	0.710
Cholesterol (mg)^b^	565.27 ± 126.35	12.05 ± 10.07	≤ 0.001^&^
Vitamin A (μg of retinol equivalents)^b^	1058.70 ± 1368.3	397.40 ± 218.70	0.006^&^
Vitamin C (mg)^b^	175.60 ± 60.92	423.40 ± 292.28	0.004^&^
Vitamin E (mg of α‐tocopherol)^b^	4.47 ± 1.63	2.77 ± 0.70	0.006^&^
Selenium (μg)^b^	87.27 ± 27.11	27.48 ± 0.02	≤ 0.001^&^
**HEI‐2015** ^**d**^			
**Components** ^**d**^	**VEGAN (*n* = 7)**	**OMN (*n* = 7)**	***p***
	**Median (IQR)**	**Median (IQR)**	
Total fruits^c^	4.70 (1.39)^1^	3.38 (2.82)^1^	0.037
Whole fruits^c^	4.53 (1.89)	2.90 (2.62)	0.102
Total vegetables^c^	4.92 (1.30)^1,2^	2.15 (1.73)^2^	< 0.001
Green vegetables^c^	4.74 (2.47)^1^	1.25 (2.45)^1,2^	< 0.001
Whole grains^c^	4.64 (6.68)^1^	1.79 (2.71)^1,2^	0.005
Dairy^c^	0.00^1,2^	1.76 (2.74)^2^	< 0.001
Total protein foods^c^	4.60 (0.89)	5.0 (0.38)^1^	< 0.001
Seafood and plant proteins^c^	5.0 (0.29)^1,2^	3.75 (1.53)^2^	< 0.001
Fatty acids^c^	8.20 (1.87)^1,2^	3.26 (2.67)^2^	< 0.001
Refined grains^c^	6.67 (4.69)	7.0 (4.05)	0.492
Sodium^c^	6.54 (2.61)	6.49 (3.49)^1^	0.023
% Saturated fats^c^	9.09 (2.50)^1^	7.83 (4.32)	0.018
Added sugars^c^	8.62 (2.81)	7.90 (4.03)	0.397
Total HEI‐2015^d^	68.31 (3.86)^1,2^	52.93 (16.91)^2^	< 0.001

*Note:* Values with numbers in common are statistically different in Kruskal–Wallis test (*p* < 0.05).

Abbreviations: %E, percent of total energy value; g, grams; g/kg, grams ingested per kilogram body mass; IQR, interquartile range; kcal, kilocalories; mg, milligrams; OMN, omnivore; VEGAN, vegan.

^a^ Student's *t*‐test.

^b^ Mann–Whitney test.

^c^ Kruskal–Wallis statistical test.

^d^ Total fruits: fruits + juices; whole fruits; total vegetables: all plant foods, except roots and tubers; green vegetables: all green‐coloured vegetables; whole grains: all grains in their entirety, including in mixed foods; dairy: dairy products, except ultra‐processed; total protein foods: all types of meats, poultry, eggs, seafood, and plant proteins, including beans; seafood and plant proteins: all seafood and plant proteins; fatty acids: (polyunsaturated + monounsaturated)/saturated; refined grains: all refined grains in the diet, consumed alone or in compound foods; sodium: total amount of sodium in the diet; % saturated fats: percent caloric contribution of saturated fatty acids in the total energy value; added sugars: percent caloric contribution of added sugars in the total energy value.

^&^ Significant difference between ONM and VEGAN from Student's *t*‐test and Mann–Whitney tests.

Protein intake differed markedly between groups. Omnivores consumed more total protein than vegans (135.11 ± 47.35 vs. 60.12 ± 15.15 g/day; *p* = 0.005), a higher percentage of energy from protein (20.48 ± 2.55 vs. 10.02% ± 2.07% of energy; *p* ≤ 0.001), and more protein relative to body mass (2.14 ± 0.63 vs. 0.98 ± 0.24 g/kg/day; *p* = 0.002). Plant protein intake was higher in vegans than in omnivores (65.08 ± 24.56 vs. 32.25 ± 8.59 g/day; *p* = 0.017), whereas animal protein intake was higher in omnivores and absent in vegans (88.73 ± 67.75 vs. 0.00 ± 0.00 g/day; *p* ≤ 0.001). Accordingly, omnivores had higher intakes of essential amino acids (49.30 ± 17.30 vs. 18.67 ± 4.16 g/day; *p* = 0.003) and branched‐chain amino acids (23.73 ± 8.25 vs. 9.26 ± 2.05 g/day; *p* = 0.005).

Total fat intake did not differ significantly between omnivores and vegans (89.89 ± 32.68 vs. 70.78 ± 10.34 g/day; *p* = 0.183), nor did the percentage of energy from total fat (28.64 ± 7.76 vs. 25.60% ± 8.01% of energy; *p* = 0.128). However, omnivores consumed more trans fat (2.00 ± 2.62 vs. 0.58 ± 0.95 g/day; *p* = 0.038), saturated fatty acids (33.46 ± 12.59 vs. 16.74 ± 2.87 g/day; *p* = 0.012), and a higher percentage of energy from saturated fatty acids (11.55 ± 2.81 vs. 6.51% ± 1.35% of energy; *p* = 0.002). Monounsaturated fatty acid intake did not differ between groups, either in grams (29.45 ± 8.78 vs. 26.16 ± 3.34 g/day; *p* = 0.381) or as a percentage of energy (10.12 ± 0.43 vs. 10.19% ± 1.89% of energy; *p* = 0.930). Vegans consumed more polyunsaturated fatty acids in grams than omnivores (22.58 ± 4.85 vs. 17.80 ± 8.77 g/day; *p* = 0.047), although the percentage of energy from polyunsaturated fatty acids did not differ significantly (8.62 ± 1.03 vs. 5.83% ± 1.32% of energy; *p* = 0.237). Omega‐3 intake was also similar between groups (1.41 ± 1.66 vs. 2.42 ± 2.32 g/day; *p* = 0.710). Regarding micronutrient intake, omnivores had higher vitamin A (1058.70 ± 1368.30 vs. 397.40 ± 218.70 μg retinol equivalents/day; *p* = 0.006), vitamin E (4.47 ± 1.63 vs. 2.77 ± 0.70 mg α‐tocopherol/day; *p* = 0.006), and selenium intakes (87.27 ± 27.11 vs. 27.48 ± 0.02 μg/day; *p* ≤ 0.001). In contrast, vitamin C intake was higher in vegans than in omnivores (423.40 ± 292.28 vs. 175.60 ± 60.92 mg/day; *p* = 0.004).

Diet quality scores are shown in Table 2. Vegans had a higher total HEI‐2015 score than omnivores [68.31 (3.86) vs. 52.93 (16.91); *p* < 0.001]. Vegans also had higher scores for total fruits [4.70 (1.39) vs. 3.38 (2.82); *p* = 0.037], total vegetables [4.92 (1.30) vs. 2.15 (1.73); *p* < 0.001], greens and beans [4.74 (2.47) vs. 1.25 (2.45); *p* < 0.001], whole grains [4.64 (6.68) vs. 1.79 (2.71); *p* = 0.005], seafood and plant proteins [5.00 (0.29) vs. 3.75 (1.53); *p* < 0.001], fatty acids [8.20 (1.87) vs. 3.26 (2.67); *p* < 0.001], sodium [6.54 (2.61) vs. 6.49 (3.49); *p* = 0.023], and saturated fats [9.09 (2.50) vs. 7.83 (4.32); *p* = 0.018]. Omnivores had higher scores for dairy [1.76 (2.74) vs. 0.00; *p* < 0.001] and total protein foods [5.00 (0.38) vs. 4.60 (0.89); *p* < 0.001]. No significant differences were observed for whole fruits [4.53 (1.89) vs. 2.90 (2.62); *p* = 0.102], refined grains [6.67 (4.69) vs. 7.00 (4.05); *p* = 0.492], or added sugars [8.62 (2.81) vs. 7.90 (4.03); *p* = 0.397].

### 3.2 Muscle Damage Markers

**FIGURE 3:**
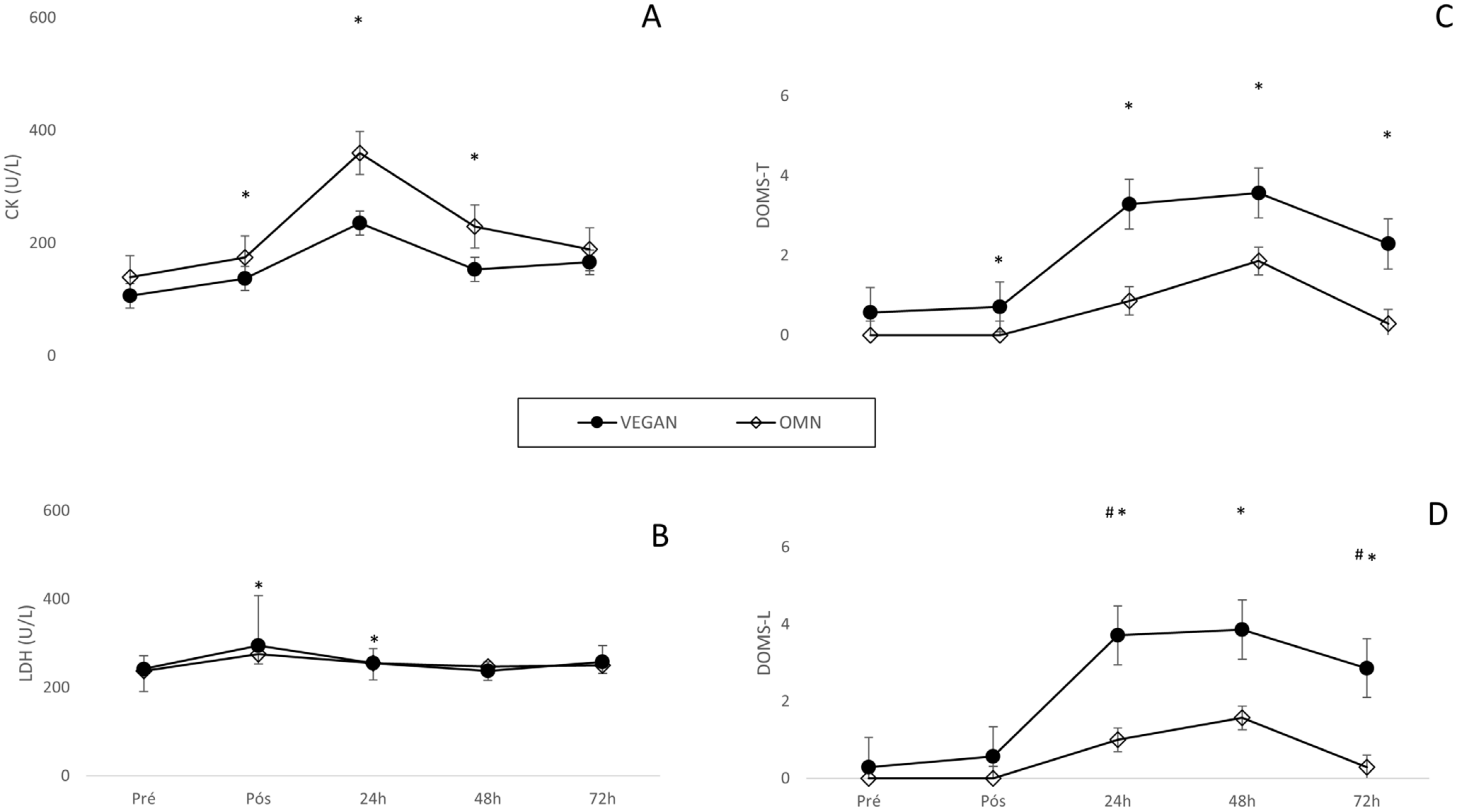
Representation plots of muscle damage markers at pre, post, 24, 48 and 72 h exercise‐induced muscle damage. Chart (A): Serum creatine kinase (CK) concentration: Significant difference between post (*p* = 0.001; ES = 0.592, small), 24 h (*p* < 0.001; ES = 0.628, moderate) and 48 h (*p* = 0.001; ES = 0.586, small) times compared to pre; Chart (B): Representation of serum lactate dehydrogenase (LDH) concentration: Significant difference between the post (*p* = 0.034; ES = 0.324, small) and 24 h (*p* = 0.046; ES = 0.292, small) times compared to the pre time; Graph (C): Representation of delayed‐onset muscle soreness with transverse palpation (DOMS‐T): Significant differences between time points 24 h (*p* = 0.002; ES = 0.580, small), 48 h (*p* < 0.001; ES = 0.619, moderate) and 72 h (*p* = 0.015; ES = 0.402, small) compared to the pre time point; Graph (D): Representation of delayed‐onset muscle soreness with longitudinal palpation (DOMS‐L): Significant differences between time points 24 h (*p* = 0.001; ES = 0.600, moderate), 48 h (*p* < 0.001; ES = 0.652, moderate) and 72 h (*p* = 0.005; ES = 0.495, small) compared to the pre time point. Differences were also observed between groups at 24 h (*p* = 0.038; ES = 0.311, small) and 72 h (*p* = 0.018; ES = 0.386, small). (*) significant difference between the time points of assessment compared to the pre time point; (#) significant difference between groups.

Muscle damage markers are presented in Figure 3 and Table S2. CK changed significantly over time after EIMD [*F*(1.872, 22.469) = 12.516; *p* < 0.001; $\eta_{p}^{2}$ = 0.511], with higher values immediately post‐EIMD, at 24 h, and at 48 h compared with baseline. In the total sample, CK increased from 122.85 ± 76.29 U/L at baseline to 155.80 ± 98.45 U/L immediately post‐EIMD, peaked at 24 h (297.51 ± 206.53 U/L), and remained elevated at 48 h (191.06 ± 113.37 U/L), before declining at 72 h (177.40 ± 129.37 U/L). No significant group effect was observed for CK [*F*(1.872, 22.469) = 1.248; *p* = 0.303; $\eta_{p}^{2}$ = 0.094]. CK values were 106.23 ± 89.50, 137.15 ± 111.22, 235.45 ± 201.77, 152.99 ± 118.53, and 165.72 ± 165.86 U/L in vegans at baseline, post‐EIMD, 24, 48, and 72 h, respectively. Corresponding values in omnivores were 139.47 ± 62.89, 174.45 ± 88.43, 359.56 ± 206.70, 229.13 ± 102.06, and 189.09 ± 91.85 U/L.

LDH also changed significantly over time [*F*(1.657, 19.882) = 3.887; *p* = 0.044; $\eta_{p}^{2}$ = 0.245], with higher values immediately post‐EIMD and at 24 h compared with baseline. In the total sample, LDH increased from 239.91 ± 37.75 U/L at baseline to 285.06 ± 78.77 U/L immediately post‐EIMD and remained elevated at 24 h (254.80 ± 34.52 U/L), before returning toward baseline at 48 h (242.18 ± 25.60 U/L) and 72 h (254.13 ± 27.96 U/L). No significant group effect was observed for LDH [*F*(1.657, 19.882) = 0.345; *p* = 0.673; $\eta_{p}^{2}$ = 0.028]. LDH values in vegans were 241.89 ± 30.11, 249.99 ± 112.86, 254.61 ± 33.84, 237.55 ± 20.33, and 258.08 ± 36.34 U/L at baseline, post‐EIMD, 24 h, 48 h, and 72 h, respectively. In omnivores, the corresponding values were 237.94 ± 46.61, 275.13 ± 21.82, 254.99 ± 37.91, 246.80 ± 30.93, and 250.17 ± 18.35 U/L.

DOMS assessed by transverse palpation changed significantly over time [*F*(2.140, 25.680) = 13.881; *p* < 0.001; $\eta_{p}^{2}$ = 0.536], with higher values at 24, 48, and 72 h compared with baseline, but no significant group effect was observed [*F*(2.140, 25.680) = 2.031; *p* = 0.149; $\eta_{p}^{2}$ = 0.145]. In the total sample, transverse DOMS increased from 0.29 ± 0.61 at baseline to 0.36 ± 0.63 immediately post‐EIMD, 2.07 ± 2.12 at 24 h, 2.71 ± 2.30 at 48 h, and 1.29 ± 1.93 at 72 h. In vegans, transverse DOMS values were 0.57 ± 0.78, 0.71 ± 0.75, 3.29 ± 2.05, 3.57 ± 2.29, and 2.29 ± 2.28 across the same time points. In omnivores, the corresponding values were 0.00 ± 0.00, 0.00 ± 0.00, 0.86 ± 1.46, 1.86 ± 2.11, and 0.29 ± 0.75.

DOMS assessed by longitudinal palpation showed a significant time effect [*F*(1.818, 21.822) = 15.238; *p* < 0.001; $\eta_{p}^{2}$ = 0.559] and a significant group effect [*F*(1.818, 21.822) = 3.747; *p* = 0.044; $\eta_{p}^{2}$ = 0.238]. In the total sample, longitudinal DOMS increased from 0.14 ± 0.36 at baseline to 0.29 ± 0.61 immediately post‐EIMD, 2.36 ± 2.46 at 24 h, 2.71 ± 2.43 at 48 h, and 1.57 ± 2.20 at 72 h. Post hoc analyses showed higher longitudinal DOMS in vegans than in omnivores at 24 h (3.71 ± 2.62 vs. 1.00 ± 1.41; *p* = 0.038) and 72 h (2.86 ± 2.47 vs. 0.29 ± 0.75; *p* = 0.018). At 48 h, longitudinal DOMS was numerically higher in vegans than in omnivores (3.86 ± 2.61 vs. 1.57 ± 1.71), but this between‐group difference was not statistically significant in the post hoc analysis.

### 3.3 Muscle Recovery Markers

**FIGURE 4:**
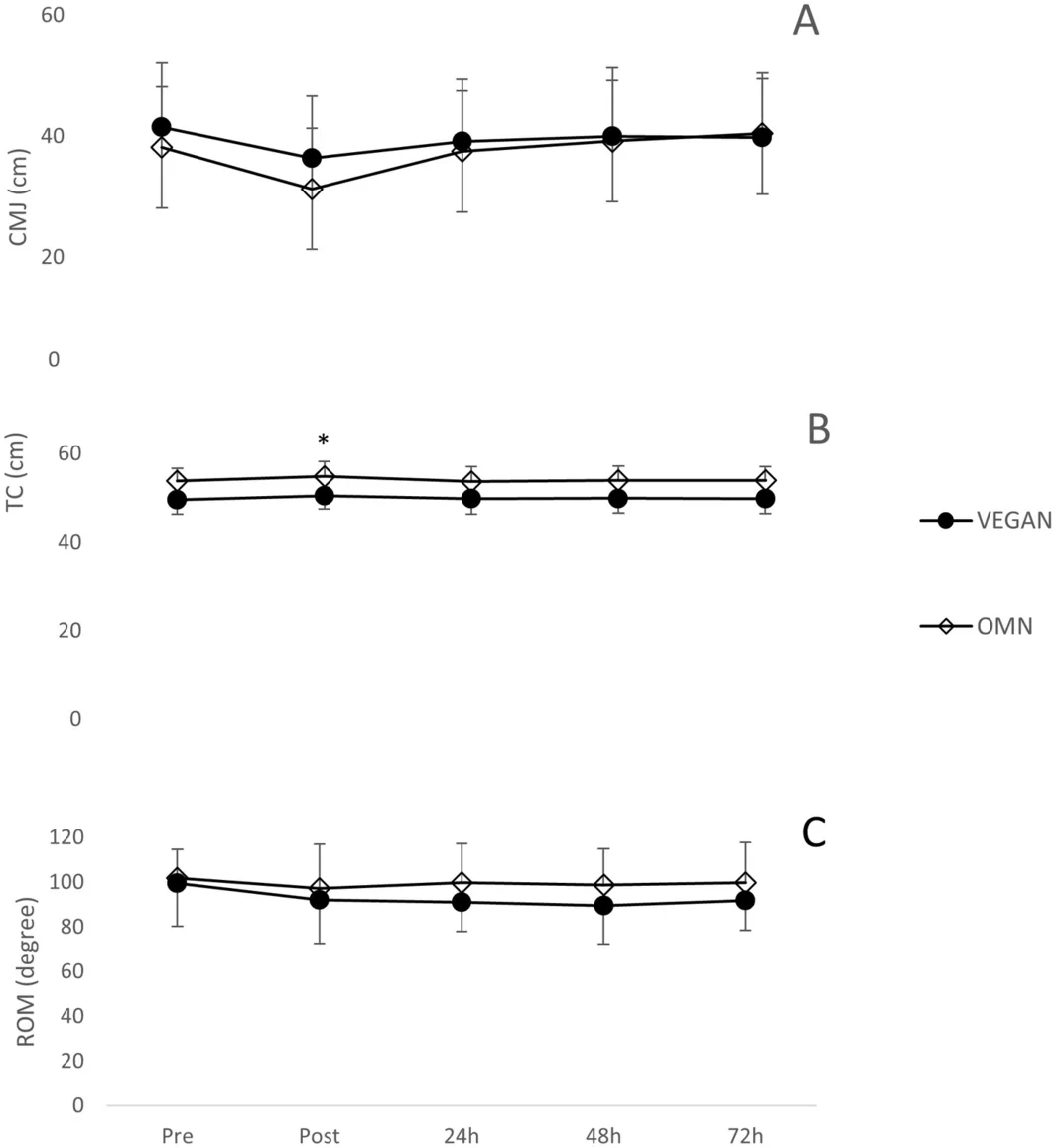
Representation graphs of muscle recovery markers at pre, post, 24, 48 and 72 h to exercise‐induced muscle damage. Graph (A): Representation of muscle power evaluated by countermovement hopping (CMJ); Graph (B): Representation of muscle swelling assessed by tight circumference (TC): There was a difference between the pre and post moments (*p* < 0.001; ES = 0.612, moderate); Graph (C): Representation of range of movement (ROM). (*) significant difference in relation to the pre moment. (#) significant difference between groups.

Muscle recovery outcomes are presented in Figure 4 and Table S3. CMJ did not show a significant time effect [*F*(4, 32) = 0.55; *p* = 0.702; $\eta_{p}^{2}$ = 0.064] or group effect [*F*(4, 32) = 1.16; *p* = 0.348; $\eta_{p}^{2}$ = 0.126]. In the total sample, CMJ decreased from 40.09 ± 12.54 cm at baseline to 34.25 ± 10.17 cm immediately post‐EIMD, corresponding to an approximate 14.6% reduction, and then returned toward baseline at 24 h (38.44 ± 10.61 cm), 48 h (39.65 ± 11.84 cm), and 72 h (40.09 ± 11.25 cm). In vegans, CMJ values were 41.47 ± 10.71, 36.36 ± 10.24, 39.12 ± 10.25, 39.99 ± 11.27, and 39.84 ± 9.68 cm at baseline, post‐EIMD, 24, 48, and 72 h, respectively. In omnivores, the corresponding values were 38.15 ± 15.88, 31.30 ± 10.41, 37.52 ± 12.25, 39.18 ± 13.95, and 40.45 ± 14.40 cm.

Thigh circumference changed significantly over time [*F*(2.624, 31.486) = 6.161; *p* < 0.001; $\eta_{p}^{2}$ = 0.339], with a significant increase immediately post‐EIMD compared with baseline. In the total sample, thigh circumference increased from 51.67 ± 3.67 cm at baseline to 52.62 ± 3.80 cm immediately post‐EIMD, corresponding to an approximate 1.8% increase, and then returned toward baseline at 24 h (51.78 ± 3.83 cm) and 48 h (51.88 ± 3.76 cm). No significant group effect was observed for thigh circumference [*F*(2.624, 31.486) = 0.393; *p* = 0.733; $\eta_{p}^{2}$ = 0.339]. In vegans, thigh circumference values were 49.54 ± 3.30, 50.42 ± 2.99, 49.84 ± 3.51, 49.85 ± 3.26, and 49.77 ± 3.41 cm across the five time points. In omnivores, the corresponding values were 53.80 ± 2.79, 54.81 ± 3.34, 53.72 ± 3.27, 53.91 ± 3.22, and 53.88 ± 3.12 cm.

ROM did not differ significantly over time [*F*(4, 44) = 1.264; *p* = 0.298; $\eta_{p}^{2}$ = 0.103] or between groups [*F*(4, 44) = 0.363; *p* = 0.833; $\eta_{p}^{2}$ = 0.032]. In the total sample, ROM was 100.64° ± 15.92° at baseline, 94.46° ± 18.84° immediately post‐EIMD, 95.08° ± 15.27° at 24 h, 93.75° ± 16.75° at 48 h, and 95.46° ± 18.09° at 72 h. In vegans, ROM values were 99.51° ± 19.13°, 92.02° ± 19.67°, 91.12° ± 13.11°, 89.42° ± 17.16°, and 91.78° ± 13.37° across the same time points. In omnivores, the corresponding values were 101.96° ± 12.84°, 97.30° ± 19.67°, 99.70° ± 17.49°, 98.80° ± 16.22°, and 99.75° ± 23.02°.

## 4 Discussion

The present study indicates that a habitual low‐protein vegan diet may be associated with greater acute post‐exercise muscle soreness, without significantly affecting overall muscle recovery following EIMD. In the present study, omnivorous participants consumed 2.14 g/kg/day of protein, whereas vegan participants consumed 0.98 g/kg/day, and the vegan group reported higher DOMS at 24 and 72 h post‐exercise. These results suggest that the observed differences might be attributable to variations in protein intake rather than solely to the consumption of animal‐ versus plant‐based protein sources.

DOMS, a subjective symptom of ultrastructural muscle injury, typically occurs following high‐intensity exercise, particularly exercise involving eccentric contractions (Wiecha et al. 2024). Its incidence is closely associated with unfamiliar physical exertion and mechanical stress (Hotfiel et al. 2018). Although considered a mild subjective symptom of injury, DOMS is a frequent cause of impaired athletic performance (Cheung et al. 2003). Importantly, the absolute differences in DOMS scores were both statistically significant and clinically meaningful, with vegans reporting higher DOMS than omnivores at both 24 h (+2.71 cm, *p* = 0.02) and 72 h (+2.57 cm, *p* = 0.01) post‐exercise in the current study. To contextualise these values, pain literature indicates that the minimal clinically important difference (Kelly 2001) for musculoskeletal pain assessed using a VAS ranges from 1 to 2 cm. Thus, the differences observed in the present study (~2.6 cm) exceeded this benchmark, suggesting a perceptible increase in muscle soreness among vegan participants. Importantly, this difference reflects subjective discomfort rather than impaired functional recovery, as CMJ, ROM, and thigh circumference did not differ significantly between groups. Given the nutritional profiles of the study groups, the most likely dietary factor explaining the observed differences in DOMS is the lower intake of total protein and amino acids in the vegan group.

Protein and amino acids are involved in muscle repair and remodelling after damaging exercise (Brown et al. 2018; Fouré and Bendahan 2017; Shenoy et al. 2016; Sousa et al. 2014; VanDusseldorp et al. 2018; Waldron et al. 2018). In a systematic review and meta‐analysis of studies on resistance EIMD, Pearson et al. (2023) reported that protein supplementation, compared with control conditions, improved isokinetic maximal voluntary contraction at 24–72 h and reduced CK concentrations at 48–72 h post‐exercise. However, the authors also noted that baseline protein intake in the included trials was generally adequate, ranging from 0.8 to 2.1 g/kg/day in protein‐supplemented groups and from 0.8 to 2.0 g/kg/day in control groups (Pearson et al. 2023). In the present study, protein intake was 2.14 g/kg/day in omnivorous participants and 0.98 g/kg/day in vegan participants. Therefore, the lower protein intake observed in the vegan group may be a plausible dietary factor related to their greater DOMS response.

In our sample, protein contributed approximately 20% of total energy intake in the omnivorous group and 10% in the vegan group. Neufingerl and Eilander (2021) reported average protein intakes of approximately 16% of energy among meat eaters and 13% among vegans across adult studies; however, their review was not specific to young, physically active Brazilian adults (Neufingerl and Eilander 2021). Thus, this comparison is used only to contextualise our findings. The relatively low contribution of protein to total energy intake in the vegan group, together with lower absolute and body‐mass‐adjusted protein intake, may be relevant to their greater DOMS response.

In addition, vegan participants consumed fewer BCAAs than omnivorous participants. BCAAs may be relevant to recovery after EIMD because they are involved in muscle protein metabolism, and recent evidence suggests that BCAA supplementation may reduce DOMS and CK after damaging exercise, although effects vary according to dose, timing, and duration of supplementation (Salem et al. 2024). The estimated daily BCAA requirement for active young adults is 337–440 mg/kg/day. This estimate is based on Indicator Amino Acid Oxidation (IAAO) studies using high‐quality isolated protein sources, such as egg or milk protein, in which BCAAs represent approximately 20% of total protein (Kaspy et al. 2024; Malowany et al. 2019; Trommelen and van Loon 2021). In our study, both groups consumed less than this benchmark: omnivores consumed 230 mg/kg/day and vegans consumed 92.6 mg/kg/day. This finding is highly relevant to the Brazilian dietary context, as much of the dietary protein comes from plant‐based sources, such as rice and beans (Rodrigues et al. 2021), which provide adequate total protein but may offer relatively lower amounts of specific BCAAs. Leucine intake was also higher in omnivorous than in vegan participants (10.45 vs. 4.18 g/day). This may be relevant mechanistically, as leucine is involved in regulating muscle protein synthesis and post‐exercise muscle remodelling (Borgenvik et al. 2012; Dreyer et al. 2008). These findings emphasise that protein quality and amino acid composition, rather than total intake alone, may be critical for muscle repair in real‐world diets.

Previous clinical trials have reported mixed findings regarding the effects of protein or amino acid supplementation on recovery after EIMD. Some studies found no significant differences between supplemented and control conditions, which may be partly explained by differences in participants' training status, supplement type and dose, protein source, timing of intake, and duration of supplementation (Jackman et al. 2010; Sousa et al. 2014). Fouré and Bendahan (2017) suggested that amino acid supplementation may be more effective when provided for several days before and/or around the damaging exercise bout, particularly when protocols last more than 10 days, rather than when supplementation is provided only acutely (Fouré and Bendahan 2017). This distinction is relevant because many trials have investigated acute protein supplementation before or immediately after EIMD, whereas the present study assessed habitual dietary intake throughout the recovery monitoring period (Betts et al. 2009; Brown et al. 2018; Cermak et al. 2009; Kephart et al. 2016). Thus, our study adds to the literature by focusing on habitual protein and amino acid intake, which may represent a more realistic and sustained nutritional exposure during the recovery period.

Regarding micronutrient intake, our findings provide an additional perspective. In the present study, vegan participants had higher vitamin C intake, whereas omnivorous participants had higher intakes of vitamin A, vitamin E, and selenium, indicating that antioxidant‐related micronutrient intake was not uniformly higher in the vegan group. A comparable study by Njeim et al. (2024), which also assessed DOMS‐related responses after an acute resistance exercise stimulus in vegans and omnivores, reported contrasting findings. In that study, 27 vegan and 27 omnivorous untrained young women were assessed before and 48 h after exercise. They reported more favourable DOMS‐related responses in vegans, mainly reflected by higher pressure pain thresholds in selected muscles at 48 h post‐exercise, while muscle circumferences were unchanged or changed similarly between groups. Although protein intake was numerically higher in omnivores than in vegans in their study, the authors suggested that the more favourable DOMS‐related response in vegans could be related to the anti‐inflammatory properties of vegan diets, potentially reflecting broader dietary differences rather than protein intake alone (Njeim et al. 2024). In contrast, our findings suggest that this potential antioxidant intake profile may not be consistent across vegan diets, particularly when antioxidant‐related micronutrient intake is heterogeneous. Because vitamin C, vitamin E, and selenium are involved in antioxidant defence and redox balance during exercise (Kim 2023; Meng and Su 2024), the heterogeneous distribution of antioxidant‐related micronutrient intake between groups should be interpreted cautiously. Therefore, in the present study, these micronutrients may not have conferred a distinct protective effect among vegan participants.

Thus, our results suggest that although the vegan group exhibited greater perceived muscle soreness—possibly related to their lower habitual protein intake, lower intake of essential amino acids and BCAAs, and some anti‐inflammatory micronutrients—no significant differences were observed between groups in acute muscle recovery. We hypothesise that this finding may be explained by several factors. First, although vegans consumed lower amounts of protein and some antioxidant‐related micronutrients, total energy intake was similar between groups (OMN: 2615 kcal; VEGAN: 2371 kcal; *p* = 0.488). This similar energy intake may have helped support acute muscle recovery, even in the context of lower habitual intake of nutrients potentially relevant to muscle repair and redox balance. When energy expenditure exceeds intake, performance and recovery may be impaired (Academy of Nutrition and Dietetics Dietitians of Canada 2016; Kerksick et al. 2018; Logue et al. 2018). However, this was not observed in our results; therefore, adequate caloric intake may have played a protective role by maintaining cellular energy status and supporting the body's capacity to recover acutely, even in the context of lower habitual intake of protective nutrients such as protein and antioxidants.

Second, we hypothesise that participants' prior training experience may have contributed to the similar functional recovery outcomes observed across both groups. In the present study, all participants were physically active, and training experience did not differ significantly between vegan and omnivorous participants. Previous exposure to resistance exercise may attenuate the magnitude of functional impairment after a subsequent exercise bout, a phenomenon commonly described as the repeated‐bout effect (Doma et al. 2023; Zourdos et al. 2015). This protective adaptation has been attributed to neural, mechanical, and cellular changes that may attenuate strength loss and help preserve functional performance after eccentric or high‐intensity exercise (Calvo‐Rubio et al. 2024). Therefore, although the vegan group reported greater DOMS at 24 and 72 h post‐exercise, their previous resistance‐training experience may have helped preserve functional responses after the EIMD protocol. This may partly explain why greater perceived soreness did not translate into significant between‐group differences in CMJ, ROM, or thigh circumference.

In addition, evidence from studies comparing vegan and omnivorous diets suggests that, when protein intake is adequate or matched between groups, vegan diets do not appear to impair strength‐related adaptations. For example, Hevia‐Larraín et al. (2021) showed that a high‐protein vegan diet promoted increases in strength similar to those observed with a high‐protein omnivorous diet after resistance training. Similarly, Monteyne et al. (2023) demonstrated that high‐protein vegan and omnivorous diets supported comparable daily myofibrillar protein synthesis rates, lean mass gains, muscle fibre hypertrophy, and strength improvements during an 11‐week resistance‐training program. These findings are not a direct explanation for the acute DOMS response observed in the present study, but they provide useful context by suggesting that vegan diets per se may not impair functional or adaptive responses to resistance exercise when protein intake is adequate. In contrast, our study assessed acute recovery after EIMD under habitual dietary conditions, in which vegan participants consumed substantially less protein than omnivorous participants. Therefore, the differences observed in the present study may be partially explained by lower protein intake among vegan participants rather than by the vegan dietary pattern per se.

To our knowledge, the present study is among the first to assess habitual nutrient intake, muscle damage, and acute recovery following EIMD in physically active vegans and omnivores. Two similar studies have recently been published, but they were conducted in untrained individuals. Presti, Mansouri, et al. (2024) and Presti, Rideout, et al. (2024) compared 16 vegetarians and 16 omnivorous participants and reported faster recovery in the omnivorous group, which had higher total protein (94.4 ± 46.9 vs. 52.4 ± 13.4 g/day), leucine (7.3 ± 3.7 vs. 4.0 ± 1.4 g/day), and essential amino acid intake, although relative protein intake did not differ significantly (1.1 ± 0.5 vs. 0.9 ± 0.2 g/kg/day). Njeim et al. (2024) compared 27 vegan and 27 omnivorous untrained healthy young women and reported more favourable DOMS‐related responses in vegans at 48 h post‐exercise. Training status is an important difference between these studies and the present study, as untrained individuals tend to show greater functional decrements after damaging exercise, whereas prior resistance‐training experience may attenuate strength loss and help preserve functional performance after EIMD (Boyd et al. 2023; Doma et al. 2023). Therefore, the present study adds a different perspective by assessing physically active vegans and omnivores with resistance‐training experience.

Notwithstanding these significant findings, our study has some limitations. Nutrient intake was estimated from dietary recalls, and we did not assess circulating biomarkers of nutritional status, such as serum vitamin B12, ferritin, vitamin D, or antioxidant markers. In addition, essential micronutrient supplements, particularly vitamin B12, were not systematically evaluated, which may have influenced individual micronutrient status independently of dietary intake. However, we assessed overall diet quality using the Healthy Eating Index (HEI‐2015), a widely accepted tool for evaluating diet quality in vegan and omnivorous populations (Parker and Vadiveloo 2019; Torna et al. 2021; Turner‐McGrievy et al. 2008). The HEI‐2015 captures overall alignment with dietary recommendations, including adequacy components related to fruits, vegetables, whole grains, protein foods, and fatty acids, as well as moderation components such as sodium, refined grains, added sugars, and saturated fats. In the present study, vegans scored substantially higher than omnivores across nearly all HEI components, reflecting better overall diet quality. This does not contradict the lower intake of selected micronutrients observed in vegans because a higher overall diet quality score reflects the overall dietary pattern and does not guarantee higher intake of every individual micronutrient. Importantly, our primary objective was to compare acute responses to EIMD between individuals following their habitual vegan or omnivorous diets, reflecting real‐world dietary practices rather than controlled feeding conditions. This ecological approach is a strength of the study because it captures how these dietary patterns are actually practiced by physically active individuals. Also, although a 30‐day washout period for dietary supplements was implemented to minimise acute effects, the potential for longer‐term physiological adaptations from prior chronic supplement use remains a consideration when interpreting the results.

Another limitation concerns sample characteristics. Although omnivorous participants had higher BMI than vegan participants, both groups remained within the normal BMI range; this is relevant because body size may influence exercise training volume, muscle damage markers, and functional recovery responses (Kim and So 2018). In addition, the unequal distribution of men and women across groups should be considered, as sex‐related differences in muscle morphology, hormonal profile, inflammation, and regeneration may influence muscle damage and recovery responses (Hunter and Senefeld 2024; Luk et al. 2025). Finally, our sample was composed of physically active young adults of both sexes, and the findings may not extend to sedentary individuals, older adults, athletes, or Brazilian adults with different habitual dietary patterns. This is relevant because protein intake in our omnivorous group was higher than usually observed in the Brazilian adult population, whereas protein intake in our vegan group was closer to values commonly reported among Brazilian adults (Fisberg et al. 2024; Neufingerl and Eilander 2021); therefore, results may differ in populations with lower omnivorous protein intake, higher vegan protein intake, different micronutrient intake, or regular supplement use.

These findings have important clinical and practical implications for the nutritional management of physically active vegans. In the present study, vegans showed greater DOMS, whereas biochemical markers of muscle damage (CK and LDH) and functional recovery outcomes (CMJ, ROM, and thigh circumference) were similar between groups. This may suggest that lower protein intake and reduced amino acid consumption are relevant factors for perceived soreness after EIMD, even when acute functional recovery is preserved. Clinically, these findings support early dietary assessment and individualised nutritional strategies to optimise protein and amino acid intake in physically active vegans. Future studies should examine whether habitual nutrient insufficiencies are associated with suboptimal recovery responses in vegan diets, ideally incorporating blood biomarkers such as serum vitamin B12, ferritin, vitamin D, and antioxidant markers.

## 5 Conclusion

This study suggests that physically active adults following a habitual vegan diet experienced greater acute perceived muscle soreness after EIMD than those following an omnivorous diet. We hypothesise that lower intakes of protein and anti‐inflammatory micronutrients may be contributing factors. It is important to note that, in our study, vegans consumed substantially less protein than omnivores, making this dietary profile relevant to the interpretation of our findings, while the omnivorous group exhibited higher protein intakes. Additionally, the lack of control for micronutrient supplementation—particularly vitamin B12—represents a significant limitation of our study, as it may have influenced the outcomes and reduced the relative impact of protein, EAA, and BCAA intake on muscle recovery. The lack of association between food groups and muscle recovery measures might be explained by chronic physiological adaptations in individuals who regularly engage in exercise and/or by similar energy intake across dietary groups. Therefore, our findings should be interpreted with caution and may not be generalisable to more common dietary patterns.

## Author Contributions

**Raquel Simões Mendes‐Netto:** conceptualisation, methodology, formal analysis, writing – original draft, writing – review and editing, supervision. **Alice Conrado de Souza:** conceptualisation; data curation; formal analysis. **Marcos da Silva Brandão:** conceptualisation; data curation. **David Lima Oliveira:** conceptualisation; data curation. **Felipe Garcez de Carvalho:** conceptualisation; data curation. **Marcela Larissa Costa:** formal analysis; writing – original draft; writing – review and editing. **Marcus Vinicius Santos do Nascimento:** investigation; methodology. **Marzo Edir da Silva Grigoletto:** project administration; supervision writing – original draft; writing – review and editing.

## Funding

This study was partly financed by Fundação de Apoio à Pesquisa e à Inovação Tecnológica do Estado de Sergipe (FAPITEC/SE: PROMOB—88881 157882/2017‐01, FAPITEC/SE: PROEF—88887) and Coordenação de Aperfeiçoamento de Pessoal de Nível Superior (CAPES—Finance Code 001).

## Conflicts of Interest

The authors declare no conflicts of interest.

## Supporting information


**Table S1:** Sports nutrition supplements considered in the present study.
**Table S2:** Muscle damage markers of vegans and omnivores at different assessment times pre and post DMIE (*n* = 14).
**Table S3:** Markers of muscle recovery in vegans and omnivores at different assessment times pre and post DMIE (*n* = 14).

## Data Availability

The databases are available from the corresponding author upon reasonable request. The data are not publicly available due to restrictions, as they contain information that could compromise the privacy of research participants.
