# Supplementary material for: Similar Muscle Recovery but Greater Soreness in Active Vegans on Low‐Protein Diets Compared to High‐Protein Omnivores Following Exercise‐Induced Damage
Source: Nutr Bull. 2026 Aug 12;51(3):389–406. doi: 10.1111/nbu.70064 (PMC13522861; doi:10.1111/nbu.70064)
Supplement: Supplementary file 1 [file NBU-51-389-s001.docx]

**Supplementary Material**

**Title****:** Similar Muscle Recovery but Greater Soreness in Active Vegans on Low-Protein Diets Compared to High-Protein Omnivores Following Exercise-Induced Damage.

Raquel Simões Mendes-Netto^1,2,3*,^ Alice Conrado de Souza¹, Marcos da Silva Brandão², David Lima Oliveira³, Felipe Garcez de Carvalho², Marcela Larissa Costa², Marcus Vinicius Santos do Nascimento^4^, Marzo Edir da Silva Grigoletto¹

**Affiliations:**

1. Postgraduate Program in Physical Education, Federal University of Sergipe. São Cristóvão, Sergipe. Brazil. raquel@academico.ufs.br (R.S.M.N); 94alicecs@gmail.com (A.C.S.); dasilvame@gmail.com (M.E.S.G.

2. Postgraduate Program in Nutrition Sciences, Federal University of Sergipe, São Cristóvão, Sergipe. Brazil. marcos.silva_22@hotmail.com (M.S.B); felipeatem10@gmail.com (F.G.C.); marcelaa.costa.nutricionista@gmail.com (M.L.C.)

3. Nutrition Department, Federal University of Sergipe, São Cristóvão, Sergipe. Brazil. davidkon@hotmail.com (D.L.O.)

4. Department of Nutrition, Tiradentes University, Aracaju, Sergipe. Brazil. marcusnascimentone@gmail.com (M.V.S.N.)

**Table S1.** Sports nutrition supplements considered in the present study.

| **Category** | **Supplement** | **Brief description** |
| --- | --- | --- |
| Protein | Protein powders (e.g. whey, soy, pea) | Concentrated sources of protein used to support muscle repair and recovery |
| Creatine | Creatine monohydrate | Enhances phosphocreatine availability during high-intensity exercise |
| Amino acids | Branched-chain amino acids (BCAAs) | Provide leucine, isoleucine and valine in isolated form |
| Amino acids | Beta-alanine | Increases muscle carnosine and may delay fatigue |
| Multi-ingredient | Pre-workout formulas | Typically contain caffeine, amino acids and other ergogenic compounds |

Sports nutrition supplements were defined as ergogenic aids intended to enhance athletic performance or support post-exercise recovery.

**Table S2**. Muscle damage markers of vegans and omnivores at different assessment times pre and post DMIE (n=14).

| Variables | | Pre | Post | 24h | 48h | 72h |
| --- | --- | --- | --- | --- | --- | --- |
| CK^†^ | Vegan | 106.23 ± 89.50 | 137.15 ± 111.22 | 235.45 ± 201.77 | 152.99 ± 118.53 | 165.72 ± 165.86 |
|  | Omn | 139.47 ± 62.89 | 174.45 ±88.43 | 359.56 ± 206.70 | 229.13 ± 102.06 | 189.09 ± 91.850 |
|  | Total | 122.85 ± 76.29 | 155.80 ± 98.45* | 297.51 ± 206.53* | 191.06 ± 113.37* | 177.40 ± 129.37 |
| LDH^†^ | Vegan | 241.89 ± 30.11 | 249.99 ± 112.86 | 254.61 ± 33.84 | 237.55 ± 20.33 | 258.08 ± 36.34 |
|  | Omn | 237.94 ± 46.61 | 275.13 ± 21.82 | 254.99 ± 37.91 | 246.80 ± 30.93 | 250.17 ± 18.35 |
|  | Total | 239.91 ± 37.75 | 285.06 ± 78.77* | 254.80 ± 34.52* | 242.18 ± 25.60 | 254.13 ± 27.96 |
| DOMS t^†^ | Vegan | 0.57 ± 0.78 | 0.71 ± 0.75 | 3.29 ± 2.05 | 3.57 ± 2.29 | 2.29 ± 2.28 |
|  | Omn | 0.00 ± 0.00 | 0.00 ± 0.00 | 0.86 ± 1.46 | 1.86 ± 2.11 | 0.29 ± 0.75 |
|  | Total | 0.29 ± 0.61 | 0.36 ± 0.63* | 2.07 ± 2.12* | 2.71 ± 2,30* | 1.29 ± 1,93* |
| DOMS l^†^ | Vegan | 0.29 ± 0.48 | 0.57 ± 0.78 | 3.71 ± 2.62# | 3.86 ± 2.61 | 2.86 ± 2.47# |
|  | Omn | 0.00 ± 0.00 | 0.00 ± 0.00 | 1.00 ± 1.41# | 1.57 ± 1.71 | 0.29 ± 0.75# |
|  | Total | 0.14 ± 0.36 | 0.29 ± 0.61 | 2.36 ± 2.46* | 2.71 ± 2.43* | 1.57 ± 2.20* |

Factorial analysis of repeated measures with post hoc; Data displayed as mean ± SD; DMIE: exercise-induced muscle damage; VEGAN= vegan; Omn = omnivore; CK: creatine kinase; LDH: lactate dehydrogenase; DOMS L: late muscle soreness with longitudinal palpation in the quadriceps muscle group; DOMS T: late muscle soreness with transverse palpation in the quadriceps muscle group;

CK:, significant difference between post (p=0.001; ES= 0.592, small), 24hours (p<0.001; ES= 0.628, moderate) and 48hours (p= 0.001; ES= 586, small) times compared to the pre time; LDH: significant difference between times post (p= 0.034; ES= 0.324, small) and 24 hours (p=0.046; ES=0.292, small) compared to time pre; DOMS T: significant differences between times 24 hours (p=0.002; ES=0.580, small), 48 hours (p<0.001; ES=0.619, moderate) and 72 hours (p=0.015; ES=0.402, small) compared to time pre; DOMS L: significant differences between time points 24 hours (p=0.001; ES=0.600, moderate), 48 hours (p<0.001; ES=0.652, moderate) and 72 hours (p=0.005; ES=0.495, small) compared to the pre time point. Differences were also observed between groups at 24 hours (p=0.038; ES=0.311, small) and 72 hours (p=0.018; ES=0.386, small). (*) significant difference between the time points of assessment compared to the pre time point; (#) significant difference between groups; (^†^) Greenhouse–Geisser correction applied due to violation of the sphericity assumption.

**Table S3**. Markers of muscle recovery in vegans and omnivores at different assessment times pre and post DMIE (n=14).

| Variables | | Pre | Post | 24h | 48h | 72h |
| --- | --- | --- | --- | --- | --- | --- |
| CMJ | Vegan | 41.47 ± 10.71 | 36.36 ± 10.24 | 39.12 ± 10.25 | 39.99 ± 11.27 | 39.84 ± 9.68 |
|  | Omn | 38.15 ± 15.88 | 31.30 ± 10.41 | 37.52 ± 12.25 | 39.18 ± 13.95 | 40.45 ± 14.40 |
|  | Total | 40.09 ± 12.54 | 34.25 ± 10.17* | 38.44 ± 10.61 | 39.65 ± 11.84 | 40.09 ± 11.25 |
| TC | Vegan | 49.54 ± 3.30 | 50.42 ± 2.99 | 49.84 ± 3.51 | 49.85 ± 3.26 | 49.77 ± 3.41 |
|  | Omn | 53.80 ± 2.79 | 54.81 ± 3.34 | 53.72 ± 3.27 | 53.91 ± 3.22 | 53.88 ± 3.12 |
|  | Total | 51.67 ± 3.67 | 52.62 ± 3.80* | 51.78 ± 3.83 | 51.88 ± 3.76 | 49.77 ± 3.41 |
| ROM | Vegan | 99.51 ± 19.13 | 92.02 ± 19.67 | 91.12 ± 13.11 | 89.42 ± 17.16 | 91.78 ± 13.37 |
|  | Omn | 101.96 ± 12.84 | 97.30 ± 19.67 | 99.70 ± 17.49 | 98.80 ± 16.22 | 99.75 ± 23.02 |
|  | Total | 100.64 ± 15.92 | 94.46 ± 18.84 | 95.08 ± 15.27 | 93.75 ± 16.75 | 95.46 ± 18.09 |

Factorial analysis of repeated measures with post hoc; Data expressed as mean ± SD; CMJ: Countermovement jump; TC: Thigh circumference; ROM: Range of motion; TC: there was a difference between the pre and post time points (p<0.001; ES=0.612, moderate); (*) significant difference between assessment times compared to the pre time point; (#) significant difference between groups.
